# Influence of Uranium on Bacterial Communities: A Comparison of Natural Uranium-Rich Soils with Controls

**DOI:** 10.1371/journal.pone.0025771

**Published:** 2011-10-05

**Authors:** Laure Mondani, Karim Benzerara, Marie Carrière, Richard Christen, Yannick Mamindy-Pajany, Laureline Février, Nicolas Marmier, Wafa Achouak, Pascal Nardoux, Catherine Berthomieu, Virginie Chapon

**Affiliations:** 1 CEA, DSV, IBEB, Laboratoire Interactions Protéine Métal, Saint-Paul-lez-Durance, France; 2 CNRS, UMR 6191, Saint-Paul-lez-Durance, France; 3 Université Aix-Marseille, Saint-Paul-lez-Durance, France; 4 IMPMC, UMR 7590, CNRS, UPMC, IPGP, Paris, France; 5 LSDRM, CEA-CNRS UMR 3299, SIS2M, Gif-sur-Yvette, France; 6 Université Nice-Sophia-Antipolis, Nice, France; 7 CNRS UMR 6543, Centre de Biochimie, Nice, France; 8 LRSAE, Université Nice-Sophia-Antipolis, Nice, France; 9 IRSN, DEI, SECRE, LRE, Saint-Paul-lez-Durance, France; 10 CEA, DSV, IBEB, Laboratoire Ecologie Microbienne Rhizosphère and Environnements Extrêmes, Saint-Paul-lez-Durance, France; 11 SEPA, AREVA NC, Bessines-sur-Gartempe, France; Laurentian University, Canada

## Abstract

This study investigated the influence of uranium on the indigenous bacterial community structure in natural soils with high uranium content. Radioactive soil samples exhibiting 0.26% - 25.5% U in mass were analyzed and compared with nearby control soils containing trace uranium. EXAFS and XRD analyses of soils revealed the presence of U(VI) and uranium-phosphate mineral phases, identified as sabugalite and meta-autunite. A comparative analysis of bacterial community fingerprints using denaturing gradient gel electrophoresis (DGGE) revealed the presence of a complex population in both control and uranium-rich samples. However, bacterial communities inhabiting uraniferous soils exhibited specific fingerprints that were remarkably stable over time, in contrast to populations from nearby control samples. Representatives of *Acidobacteria, Proteobacteria*, and seven others phyla were detected in DGGE bands specific to uraniferous samples. In particular, sequences related to iron-reducing bacteria such as *Geobacter* and *Geothrix* were identified concomitantly with iron-oxidizing species such as *Gallionella* and *Sideroxydans*. All together, our results demonstrate that uranium exerts a permanent high pressure on soil bacterial communities and suggest the existence of a uranium redox cycle mediated by bacteria in the soil.

## Introduction

Uranium occurs naturally at high concentrations in some minerals such as uraninite or autunite but can also be disseminated in the environment as a consequence of mining activities or radioactive wastes leaching. A better understanding of the factors governing uranium bioavailability, and ultimately its potential incorporation into the food chain, are therefore of great importance. Bacterial communities in soils are considered as one of the most important factors that can influence (directly or indirectly) metal speciation and transport in the environment. Indeed, the biotransformation of uranium by a number of isolated bacteria has been well-documented in the literature. Uranium occurs environmentally in two oxidation states: the oxidized form U(VI) and the reduced form U(IV). Bacteria can modify the redox state of uranium either by reduction, leading to the formation of insoluble U(IV), or by oxidation into U(VI) and subsequent solubilisation of the metal. Reduction processes have been described in a number of species, including iron-reducing bacteria [1]–[4]. On the other hand, oxidation of uranium can be catalyzed by some iron-oxidizing bacteria [5]–[6]. Bacteria can also mediate uranium immobilization through several mechanisms including biosorption (adsorption and precipitation), and intracellular accumulation (reviewed in [7]).

Since environmental bacteria can interact with uranium to modify its speciation and mobility, uranium could in turn influence the structure and activity of bacterial communities. Such interactions have been extensively studied in environments contaminated by anthropogenic uranium. Analyses of microbial diversity in contaminated environments have been conducted by culture-dependent and -independent approaches. Numerous data have been obtained from sediment and groundwater samples collected at the Oak Ridge Field Research Center, contaminated during uranium extraction for nuclear weapons production [8]–[11]. Milling and mining impacted environments have also been analyzed in the USA and Europe [12]–[16]. Several studies have monitored changes in indigenous bacterial communities during uranium reduction and bioremediation by *in situ* biostimulation, [17]–[28], or after the addition of U(VI) to environmental samples [30]–[31]. Nevertheless, little is known to date about indigenous soil microbial communities that inhabit natural uranium ores. The study of bacterial populations subjected to long-term uranium exposure in such natural environments could thus provide a unique opportunity to identify bacterial species which have adapted to the presence of high uranium content. To address this aim, we conducted an in-depth analysis of soil samples collected in the region of Bessines (Limousin, France), one of the most important natural uranium deposits in France. This location afforded us the possibility to select two sets of samples exhibiting similar mineralogical and chemical characteristics, but with contrasting uranium content. We assessed the impact of uranium on the bacterial communities by a comparative analysis of community structures using a culture-independent method (DGGE). Our results indicate the presence of complex communities in the uraniferous samples, which are more stable over time than in controls. We also showed that uranium-rich samples host a specific population, characterized by the co-occurrence of iron-reducing bacteria related to *Geobacter*, *Geothrix* and *Acidobacteria*, as well as iron-oxidizing species related to *Gallionella* and *Sideroxidans*.

## Results

### Chemical and mineral characteristics of soil samples

**Figure 1:**
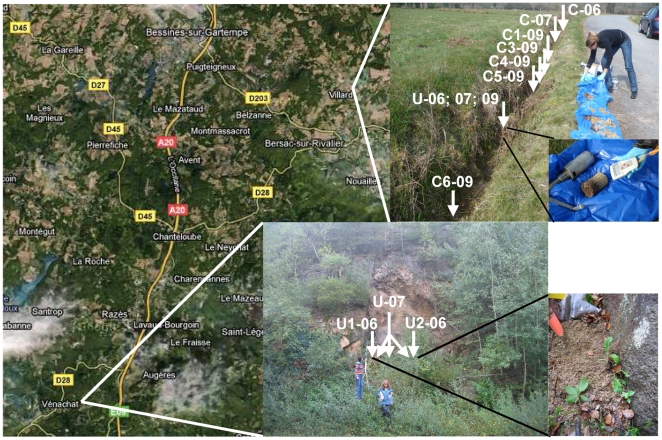
Location of the study sites Vénachat and Villard and the different sampling points. C: control samples. U: uranium-rich samples. Sampling years are labelled as: -06 (2006); -07 (2007); and -09 (2009).

Replicates of soil samples were collected from two sites, Villard and Vénachat, located near Bessines-sur-Gartempe, as detailed **in** Figure 1 and below in the Materials and Methods section. For each site, radioactive soil samples (U samples) and nearby non-radioactive controls (C samples) were subjected to chemical and mineralogical analyses.

Results for major element chemistry, selected trace elements, pH, and Total Organic Carbon (TOC) analyses are compiled in Table 1. For each series (Vénachat or Villard), TOC content, pH, major elements as well as trace elements (As, Cd, Co, Cr, Cu, Ni, Pb and Zn; not shown) were within the same range between the U and C samples, except for CaO in Vénachat U2-06. As expected, higher uranium concentrations were measured in U samples than in C samples. In Vénachat, C samples exhibited less than 100 ppm of uranium while U samples were 71–87 times more concentrated with values reaching 4,700 ppm. In Villard, the contrast was even greater: the uranium content in C samples, averaging 45 ppm, was 1,000 to 10,000 times lower than in U samples, in which uranium ranged from 14,860 to 255,000 ppm. These high uranium contents are associated with high P_2_O_5_ concentrations, which is consistent with the mineralogical nature of the soil (see further).

**Table 1:** Physicochemical characteristics of the soil samples.

Site	Sample	SiO_2_	Al_2_O_3_	Fe_2_O_3_	P_2_O_5_	CaO	Na_2_O	K_2_O	TOC	pH	CFU/g soil (×10^5^)	Total uranium (ppm)	Soluble Uranium (µM)
Vénachat	U1-06	71	16.79	2.51	0.62	0.31	3.2	4.1	-	4.6	4.2	2,617	-
	U2-06	63	17.26	2.27	0.81	4.31	4.97	4.07	0.52	4.6	2.2	2,140	-
	U-07	60.1	17.5	3.1	0.77	0.44	2.94	4.38	1.38	4.4	-	4,718	21
	C-06	-	-	-	-	-	-	-	0.56	4.9	2.2	30	-
	C-07	70.8	15.5	1.42	0.51	0.42	4.09	2.76	1.35	4.8	-	71	0.72
Villard	U-06	-	-	-	-	-	-	-	0.43	4.7	-	37,768	-
	U-07	58.62	18.06	2.13	1.6	0.2	0.51	5.45	0.17	3.8	6.8	14,860	81
	U-09	61.42	18.54	2.68	0.87	0.18	0.43	5.23	0.48	4	9	255,000	-
	C-06	-	-	-	-	-	-	-	0.61	4.7	-	27	-
	C-07	64.8	18.01	2.88	0.08	0.12	1.82	5.26	0.29	3.8	5	74	0.1
	C1-09	60.33	21.73	2.8	0.06	0.17	1.62	6.55	0.12	4.5	11.7	<30	-
	C3-09	59.46	20.68	2.55	0.13	0.18	0.98	5.75	0.25	4.5	15	<30	-
	C4-09	62.26	20.96	2.43	0.08	0.25	2.25	5.51	0.45	4.8	16.9	<30	-
	C5-09	67.54	18.5	2.34	0.07	0.13	0.86	5.33	0.13	4.8	20.7	124	-
	C6-09	69.16	16.92	2.09	0.07	0.17	1.9	4.89	0.15	4.8	21.9	<30	-

Major elements and TOC are given in weight percent. Soluble uranium was determined after three successive lixiviations with water.

-: not determined.

**Figure 2:**
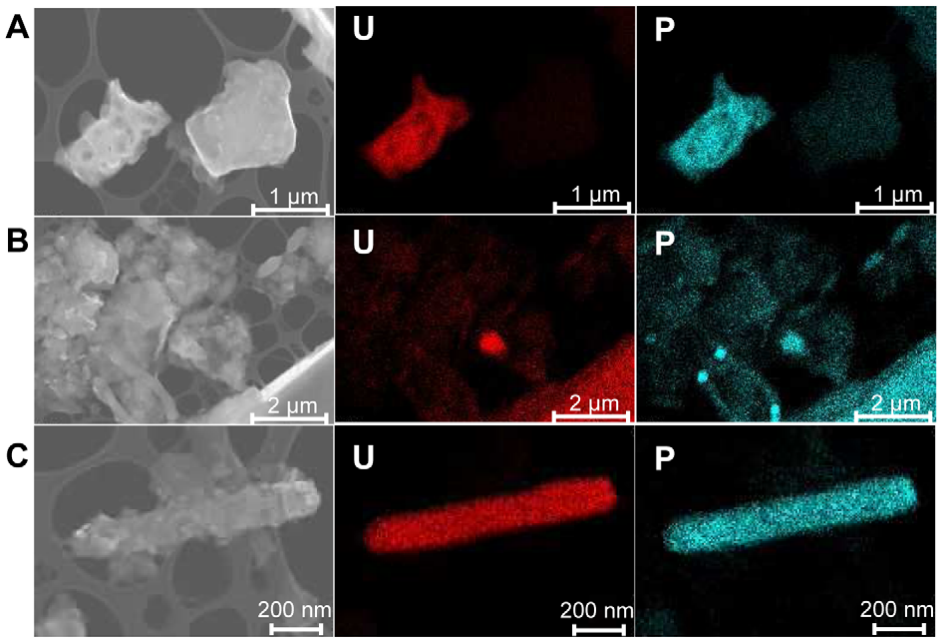
Scanning Electron Micrographs coupled with Energy Dispersive X-ray spectra analysis of Villard ViU-09 soil particles. For each SEM image, the corresponding EDXS map for uranium (U) and phosphate (P) is presented. (A) soil particles. (B) soil particles and cell-shaped objects. (C) cell-shaped object.

We determined the mineralogy of the soil samples using X-ray Diffraction (XRD). Analyses of Vénachat C-07 and U-07 samples, as well as Villard C-07 and U-07 samples were performed. All soil samples are mostly composed of quartz, orthoclase (K-feldspar), albite (Na-feldspar) and muscovite (data not shown). In Vénachat U-07, a small XRD peak at 8.40 Á is likely related to the (001) reflection of meta-autunite (Ca(UO_2_)_2_(PO_4_)_2_ · 4(H_2_O)). In Villard U-07, XRD peaks at 8.40, 5.37 and 3.61 Á, indexed as the (001), (101) and (102) planes of meta-autunite could be observed. Additionally, peaks at 9.59, 4.85 and 3.47 Á were interpreted as the (002), (004) and (200) reflections of sabugalite (HAl(UO_2_)_4_(PO_4_)_4_ · 16(H_2_O)), a common secondary mineral formed in the oxidized zone of uranium veins. These results were confirmed by Scanning Electron Microscopy coupled with Energy Dispersive X-ray Spectroscopy (SEM-EDXS) analyses that detected mineral grains containing P and U on Villard U-09 sample (Figure 2A). We also observed the presence of bright yellow particles in the uranium-rich soil from Villard, a color frequently observed in the autunite group of minerals. In addition to the high P and U contents measured by bulk chemical analyses, these data suggest that a fraction of uranium is under the form of crystalline meta-autunite and associated secondary alteration phases such as sabugalite.

**Figure 3:**
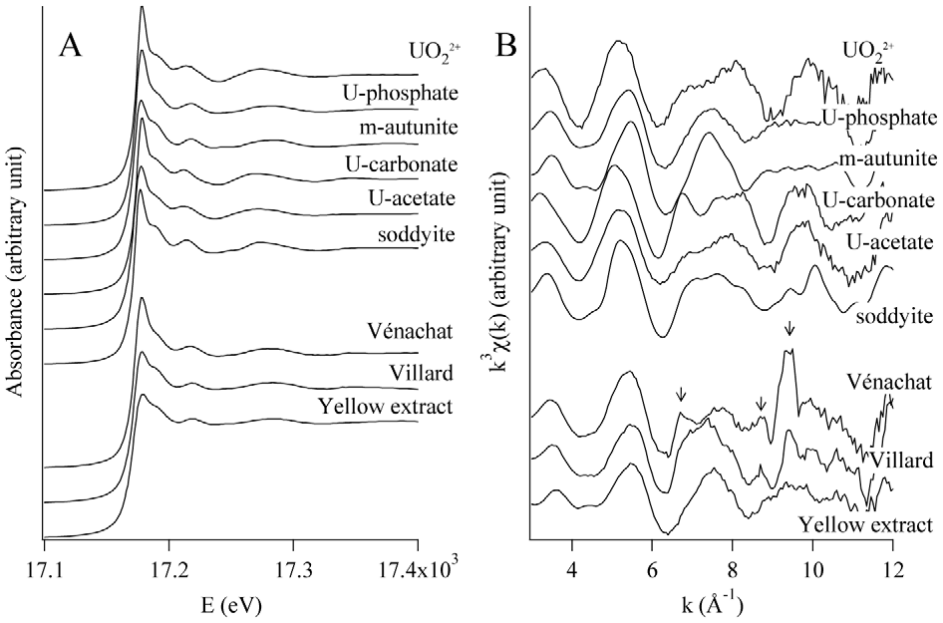
Uranium speciation in soils. (A) Uranium L_III_-edge XAS spectra, normalized to equal intensity at 17.176 keV. (B) k^3^-weighted EXAFS curves for Vénachat and Villard soils, and for yellow particles extracted from Villard soil. All spectra are compared to reference. The m-autunite spectrum was from [57].

Uranium speciation in the soils was further investigated by X-ray Absorption Spectroscopy (XAS) analysis. Spectra of Vénachat and Villard U-07 soil samples as well as yellow particles extracted from Villard soil were recorded at the U L_III_-edge (Figure 3). The position of the absorption edge at ∼17.177 keV is characteristic of the U(VI) oxidation state [31], and the shoulder at 17.188 keV in the XANES region (Figure 3A) is characteristic for the linear uranyl dioxo cation [32]. Thus, the major chemical forms of uranium in both soils and in the yellow extracts are complexes of uranyl cation.

Extracted EXAFS spectra (Figure 3B) of Vénachat and Villard soils display very different features in the region 6–8 Å^−1^, indicating different uranium speciation. EXAFS spectra of Villard soil and of yellow extracts collected from this soil are quite similar. The best result from shell-by-shell fitting applied to the EXAFS spectrum of these yellow clusters (Figure S1) is given in Table 2. These data are in the range of previously reported values for uranyl-phosphate complexes [32], and may correspond to meta-autunite and/or sabugalite (structural similarities among minerals in the uranyl-phosphate group make EXAFS spectra difficult to distinguish). A contribution at 6.5–7 Å^−1^ was observed in the EXAFS spectrum in soil from Villard but not in the spectrum of yellow extracts. This suggests the presence of another U-containing phase in this sample, which may be non-crystalline. Attempts to identify this compound by linear combination least-square fitting of the ViU-07 EXAFS spectrum, using uranyl ion, meta-autunite, sabugalite, U-carbonate, and U-acetate as references were unsuccessful.

**Table 2:** EXAFS fit results for yellow clusters extracted from Villard soil.

Sample	shell	N	R(Å)	σ^2^(Å^2^)
Villard cluster	U-Oax	2*	1.787(0.006)	0.006(0.001)
	U-Oeq	3.468(0.255)	2.241(0.005)	0.006(0.001)
	U-P	3.190(1.493)	3.673(0.117)	0.030(0.019)
	MS1	8*	3.725(0.048)	0.001(0.001)
	MS2	4*	3.810*	0.003*
	U	5*	5.061(0.184)	0.027(0.031)
m-autunite^a^	U-Oax	2	1.774(0.006)	0.003(0.001)
	U-Oeq	4	2.277(0.006)	0.004(0.001)
	U-P	4	3.59(0.002)	0.005(0.001)
	MS1	8	3.71(0.002)	0.002(0.002)
	MS2	4	3.81*	0.003*
	U	4	5.210(0.110)	0.014(0.007)

N: coordination number, R: radial distance, σ^2^: Debye-Waller factor. ΔE0 was estimated at 5.733(0.834) for the whole set of shells, and R-factor was 0.027 for the fit of Villard cluster spectrum. The estimated standard deviations are listed in parenthesis.

*: parameter fixed during fitting. MS1: U-Oeq-P multiple scattering path, MS2: U-Oeq-P-Oeq multiple scattering path.

a Data published in [32].

The EXAFS spectrum of Vénachat U-07 is more complex than that of Villard U-07. Qualitatively, two structures at 5.4 and 7.4 Å^−1^ signal the presence of a U-phosphate complex, likely the meta-autunite identified by XRD analyses. A second major contribution with beats at 6.6, 8.6 and 9.3 Å^−1^ (Figure 3B, arrows) could not be fitted properly in the EXAFS spectrum using linear combination least-square fitting. This excludes carbonate-based, silicate, or carboxylate-based species. Since meta-autunite and sabugalite were the only crystalline phases identified using XRD in this soil, the specific features observed in the EXAFS spectrum may come from a non-crystalline uranium-containing compound.

These results suggest that uranium was partly associated with insoluble mineral phases in the soils, raising the question of its bioavailability. To estimate the uranium threshold concentration to which bacterial populations are exposed *in situ*, the soluble uranium fraction was measured. For Villard and Vénachat samples, approximately 0.1% of the total uranium content was recovered in the soluble fraction after three successive lixiviations with water (Table 1).

### Microscopic observations of ViU-09 soil particles

Villard U-09 contains the highest uranium grade (25% w/w), and we chose this sample to identify uranium-rich particles and potentially uranium-associated cells. *In situ* observations by means of SEM-EDXS analysis revealed small particles that contain uranium and phosphate (Figure 2A), as well as carbon-containing objects similar to bacteria in shape and size (Figure 2B), likely corresponding to individual or filamentous bacterial cells. Several cell-shaped objects were entirely covered by particles identified as uranyl-phosphate precipitates by EDXS (Figure 2C).

Taken together, the analyses of the Villard and Vénachat soil samples indicate that the uranium-rich and control samples differ essentially in their contrasted uranium content, which is linked to the presence of mineral phases such as meta-autunite and sabugalite, and that bacteria are exposed to uranium *in situ*. Thus, comparing the natural environment of these two sites is ideal for estimating the impact of uranium on bacterial communities by a comparative study.

### Bacterial community analysis

#### 1. Vénachat

**Figure 4:**
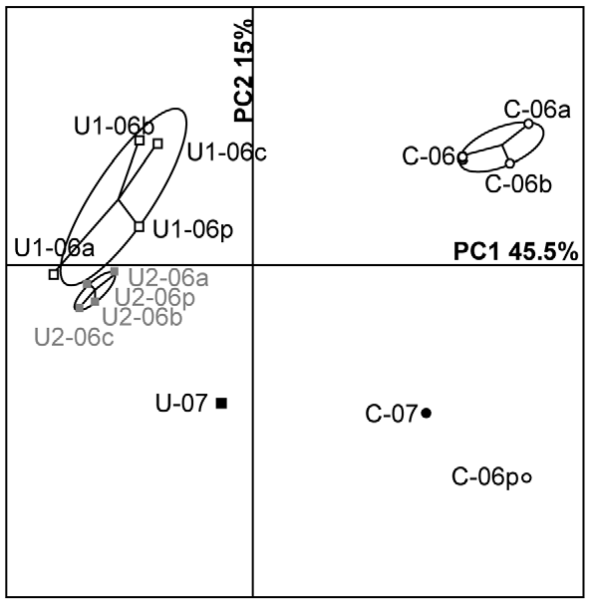
Principal Component Analysis of DGGE profiles from Vénachat soil samples. Individual sample projections are shown on the first two axes of the analysis. The first axis (PC1) and the second axis (PC2) account for 45.5% and 15% of the variability, respectively. C corresponds to control samples, whereas U, U1 and U2 correspond to uranium-rich samples. 06 and 07 indicate the years 2006 and 2007. a,b,c correspond to replicates, and p signifies pooled DNA.

Two series of samples were collected from the Vénachat site. In October 2006, uraniferous samples were collected on the soil surface at 2 different positions (designated U1-06 and U2-06), as well as controls (designated C-06) from the vicinity (see Materials and Methods). In October 2007, uraniferous (designated U-07) and control (designated C-07) samples were collected randomly from the same control and uraniferous areas and pooled. In 2006, the aerobic bacterial population cultured from the samples varied from 2.2×10^5^ to 4.2×10^5^ CFU g^−1^ of soil indicating that no significant differences were observed between uranium-rich and control soils (Table 1). PCR-DGGE was performed with universal primers targeting the V3-V5 region of 16S rRNA genes in order to compare bacterial communities inhabiting uraniferous soils (samples U1-06a, b, c, p; samples U2-06a, b, c, p; sample U-07) and control soils (samples C-06a, b, c, p; sample C-07). All samples displayed high diversity, with 30–40 discernable bands on the DGGE profiles (data not shown). Principal Component Analysis (PCA) was used to analyze the DGGE fingerprints of the soil samples. On the PC1 axis, accounting for 45.5% of the total variability, all samples corresponding to bacterial communities from uraniferous soils had clearly separated from the samples corresponding to control soils (Figure 4). Some dispersion was also observed on the PC2 axis among either control soil samples or the uranium-rich soils (representing 15% of the total variability). Although the DGGE profiles differ to some extent between sampling sites (for example, U1 *vs* U2) and sampling date (2006 *vs* 2007), their variability is not as important as that observed between uranium-rich and control samples.

#### 2. Villard

**Figure 5:**
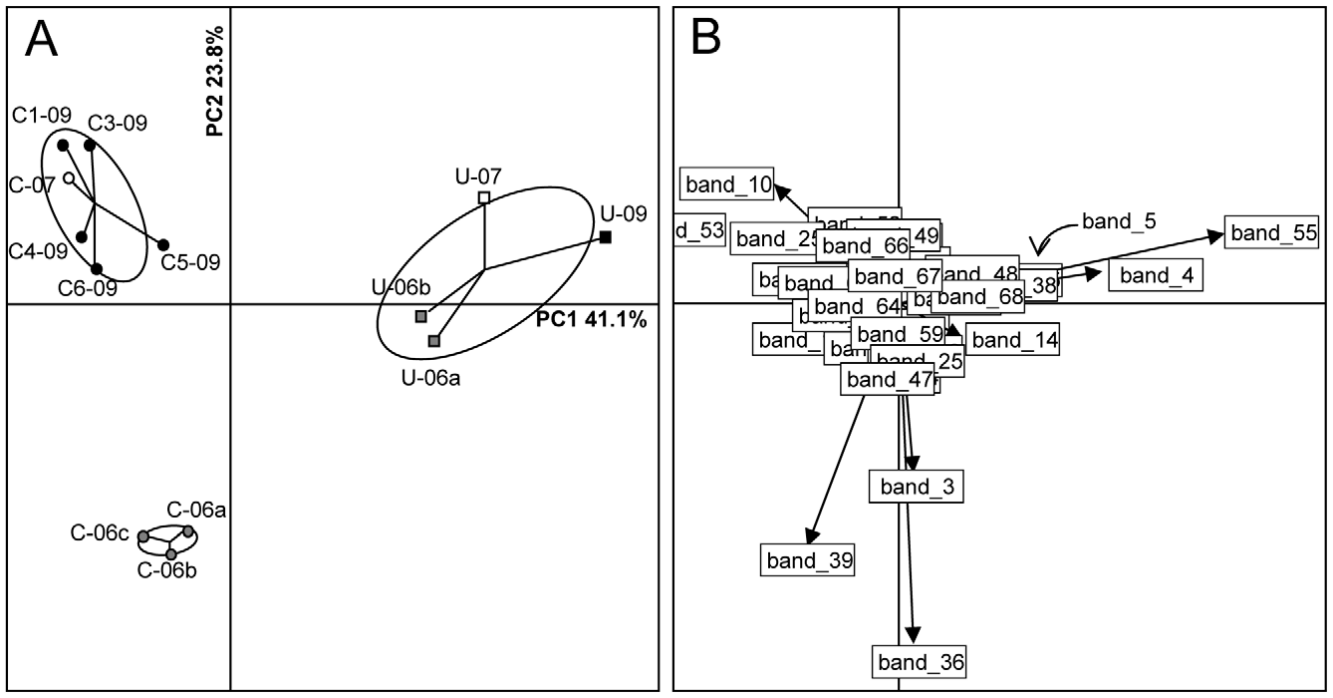
Principal Component Analysis of DGGE profiles from Villard soil samples. (A) Individual sample projections on the first two axes of the analysis. The first axis (PC1) and the second axis (PC2) account for 41.1% and 23.8% of the variability, respectively. (B) Individual DGGE band projections on the first two axes. Band_5 position (masked by band_38) is indicated with an arrow. C corresponds to control samples, whereas U corresponds to uranium-rich samples. 06, 07 and 09 indicate the years 2006, 2007 and 2009 respectively. a,b,c correspond to replicates.

Natural uraniferous and control soil samples were collected at Villard in October 2006, October 2007 and April 2009 for analysis of bacterial communities. As observed in Vénachat, the aerobic bacterial populations cultured from the U and C samples were within the same range (from 5×10^5^ to 21.9×10^5^ CFU g^−1^ of soil; Table 1). Comparisons of bacterial communities were performed by PCR-DGGE, using universal bacterial primers as described in Materials and Methods. Four uraniferous samples were processed: two collected in 2006 (U-06a and b), one in 2007 (U-07) and one in 2009 (U-09). Nine control samples, corresponding to three samples collected from the same location in 2006 (U-06a, b and c), one in 2007 (C-07) and five at different locations in 2009 (C1-09, C3-09, C4-09, C5-09 and C6-09) were included in the analysis. The bacterial communities exhibited high diversity in all soil samples, as demonstrated by the presence of multiple distinct bands on the gel (25–40 bands; data not shown). We subsequently analyzed the DGGE profiles using PCA. The first two principal components accounted for 64.8% of the variation between samples (Figure 5A). Uraniferous and control soil bacterial communities were clearly separated on the first axis, which accounts for 41.1% of the total variability. The uraniferous samples were plotted within the same statistical ellipse representing 90% confidence, whereas the controls were more dispersed. Control samples collected in 2006 were separated from the others on the second axis, accounting for 23.8% of the variability.

Figure 5B shows the distribution of the DGGE bands on the two PCA axes. Bands plotted to the right of the PC1 axis are correlated with the bacterial populations in uranium-rich soils; and reciprocally, bands plotted to the left of this axis are associated with control soil communities. Subsequently, to identify the uranium-specific population, the six DGGE bands with the greatest values on the PC1 axis (n° 4, 5, 14, 38, 55 and 68) were excised from the gel. Direct sequencing of these bands indicated that they correspond to heterogeneous sequences. To circumvent this problem, DNA fragments contained within the bands were cloned, and a total of 144 individual clones were sequenced. Phylogenetic analyses were then performed on the cloned sequences (complete data are provided in Table S1 and Figure S2). Nine bacterial phyla were detected among the sequences, which are largely represented by *Proteobacteria* and *Acidobacteria* (60 and 56 clones out of 144, respectively) (Table S1).

Among the *Proteobacteria* phylum, we detected sequences related to the four classes Alpha, Beta, Gamma and Delta. In particular, sequences were closely related to *Geobacteraceae*, a family having Fe(III)- and U(VI)-reducing members (Table 3). We also retrieved sequences closely related to *Gallionellaceae*, a family of iron-oxidizers that includes *Gallionella* and *Sideroxydans* (Table 3). 70% of the proteobacterial sequences have multiple close neighbors, found in databases, which have been reported from either uranium-contaminated or iron-rich environments (see table S1, last two columns). In particular, two *Pseudomonas* sequences, detected in DGGE band n° 5, had 63 and 71 neighbors retrieved from uranium-contaminated samples worldwide (USA-TN, -WA, -UT, Germany, Portugal, Bulgaria and India; Table 3).

**Table 3:** Phylogenetic affiliation of selected 16S bacterial sequences.

	Closest relatives (Silva 104)					Number of neighbours >95% in EMBL database	
DGGE band	similarity (%)	Accession number	Taxonomy			with uranium	with iron
B55_U07_18	94.7	HQ114173	Acidobacteria	DA023	uncultured bacterium	-	-
B55_U09_02	99.5	EU937874	Acidobacteria	DA023	uncultured bacterium	17	4
B55_U07_12	99.5	HM062339	Acidobacteria	DA023	uncultured bacterium	-	-
B55_U09_13; 20	92.3	EU297423	Acidobacteria	DA023	uncultured bacterium	-	-
B5_U07_03	91.1	FJ004730	Acidobacteria	DA023	uncultured bacterium	-	-
B38_U07_13	98.5	HM061965	Acidobacteria	DA023	uncultured bacterium	-	-
B55_U07_13; B4_U09_03	98.9-98.7	FJ466151	Acidobacteria	DA052	uncultured bacterium	2	-
B55_U07_02; 04; 07; 08; 11; 14	99.8-99.1	EU335357	Acidobacteria	DA052	uncultured bacterium	6	2
B55_U09_07; 10; 11	99.6-99.1	EU335357	Acidobacteria	DA052	uncultured bacterium	6	2
B55_U06_04; 07	99.6	EU335357	Acidobacteria	DA052	uncultured bacterium	6	2
B55_U09_01	98.3	FJ004659	Acidobacteria	DA052	uncultured bacterium	-	-
B55_U07_01	99.1	DQ451504	Acidobacteria	DA052	uncultured bacterium	5	2
B55_U07_16	98.9	EU680444	Acidobacteria	DA052	uncultured bacterium	7	2
B55_U07_19	99.6	EF018280	Acidobacteria	DA052	uncultured bacterium	6	2
B4_U09_02; 16	99.1-98.2	GQ342323	Acidobacteria	Holophagaceae/Geothrix	uncultured bacterium	8	4
B14_U07_16	98.7	GQ342323	Acidobacteria	Holophagaceae/Geothrix	uncultured bacterium	8	6
B14_U07_3a	97.5	FR667839	Betaproteobacteria	Gallionellaceae/Sideroxydans	uncultured bacterium	-	6
B4_U07_E11	98.9	AJ582038	Betaproteobacteria	Gallionellaceae/Sideroxydans	uncultured bacterium	1	74
B4_U09_08	99.8	AJ582038	Betaproteobacteria	Gallionellaceae/Sideroxydans	uncultured bacterium	2	76
B38_U07_05	94	AB240323	Betaproteobacteria	Gallionellaceae	uncultured bacterium	-	-
B14_U07_04	96.2	AB240324	Betaproteobacteria	Gallionellaceae	uncultured bacterium	-	17
B4_U07_E16	93.3	DQ450774	Betaproteobacteria	Gallionellaceae	uncultured bacterium	-	-
B5_U07_2f	99.3	DQ778036	Gammaproteobacteria	Pseudomonadaceae	Pseudomonas sp.AD21	71	82
B5_U07_18	100	GU569131	Gammaproteobacteria	Pseudomonadaceae	uncultured Pseudo. sp.	63	81
B5_U07_12	91.3	GU270815	Deltaproteobacteria	Geobacteraceae	uncultured bacterium	-	-
B14_U07_05	91.3	AY607189	Deltaproteobacteria	Geobacteraceae	uncultured Geobacter	-	-
B4_U09_13	97.8	FR667779	Deltaproteobacteria	Geobacteraceae	uncultured bacterium	9	17
B5_U07_09	98.7	CP001124	Deltaproteobacteria	Geobacteraceae	Geobacter bemidjiensis	10	6

The sequences derived from DGGE bands characterizing uranium-rich soil samples from Villard. The number of close relatives (having >95% sequence similarity), detected in uranium-contaminated or iron-rich environments is indicated in the two last columns.

The *Acidobacteria* phylum was also largely represented in DGGE bands characteristic of uranium-rich soils. Close relationships were identified with other uranium-contaminated or iron-rich environments. A group of 17 sequences, closely related to an uncultured clone from an iron(II)-rich seep (DA052, [33]), was recovered from band n° 55 excised from 2006, 2007 and 2009 DGGE profiles. Sequences related to the family *Holophagaceae* and the genus of iron-reducers *Geothrix* were also detected. These sequences had close neighbors identified in uranium-contaminated and iron-rich environments (Table 3).

Seven other phyla were also detected at lower frequencies: *Chloroflexi*, *Firmicutes*, *Nitrospirae*, *Actinobacteria, Deinococcus-Thermus, Elusimicrobia* and *Verrumicrobia* (Table S1). Two sequences had 17 close neighbors in uranium-contaminated environments: one sequence was affiliated to *Microbacterium* and the other to *Ktedonobacterales*. Finally, one sequence related to the radio-resistant *Deinococcus* genera was detected in DGGE band n° 14.

## Discussion

In our study of Vénachat and Villard soils, several factors promoted an ideal situation in which to investigate the effect of uranium on bacterial populations inhabiting aerobic soils *in situ*. First, we were able to collect samples with very high uranium content (2,140–255,000 mg.kg^−1^); by comparison, previous studies of uranium-contaminated sediment or groundwater have reported a more limited range of a few hundred mg.kg^−1^ or mg.l^−1^ [9], [13]–[15], [17], [20], [22], [29]. And while we detected uranium in control samples, these concentrations only ranged from 45 to 70 ppm (i.e., 80 - 10,000 times lower than in uranium-rich samples). Second, chemical and XRD analyses showed that for each site, uranium-rich and control samples collected in close vicinity had comparable chemical and mineralogical characteristics. XANES, EXAFS and EDX analyses demonstrated that uranium is present as uranyl associated predominantly with phosphate. XRD data highlight the presence of two insoluble forms of uranium, meta-autunite and sabugalite, raising the question of its bioavailability. However, lixiviation experiments indicate that 0.1% of uranium was water soluble, corresponding to concentrations of 21 and 81 µM in soils from Vénachat and Villard, respectively. These concentrations, which probably underestimate the amount of uranium to which bacteria are really exposed, were within the range of the total uranium content reported in uranium-contaminated samples analyzed in the literature (e.g. in [8], [12], [14], [15], [17], [21]). Observation of uranium-coated cells on Villard soil particles provided further evidence that bacteria are exposed to uranium *in situ*. Future studies using synchrotron-based scanning transmission x-ray microscopy will be interesting to perform in order to detect the possible incorporation of organic matter in uranium precipitates at the submicrometer sale and tackle a potential biotic origin (e.g. in [34]).

XAS and XRD analysis demonstrated the presence of insoluble forms of uranium phosphate mineral phases (meta-autunite, sabugalite) in the soils. In addition, some cells are entirely covered by uranium phosphate precipitates as showed by SEM/EDX analysis. Together, these results suggest a possible role of environmental bacteria in the formation of mineral phases of uranium in the soils by a biomineralization process. This hypothesis is consistent with some results reported in several studies showing that bacteria can mediate the formation of meta-autunite like phases, in acidic pH conditions comparable to those of the present study. For example, the biomineralization of uranium has been evidenced in the model bacteria *Myxococcus xanthus* [35] as well as in different environmental isolates such as *Microbacterium, Sphingomonas* and *Rahnella* [36]–[38].

DGGE provides a rapid and efficient means to investigate soil bacterial communities with the aim of detecting changes in community structures. This approach also offers the possibility to compare numerous samples. This technique was thus used to estimate the impact of uranium on soil bacterial communities. The number of bands observed on the DGGE profiles reveals a broad phylogenetic diversity in uraniferous soil samples, despite the presence of elevated amounts of uranium. Furthermore, the number of culturable isolates was not reduced by the uranium content, indicating that uranium does not reduce the number of living cells in the soil samples. The diversity of soil bacteria observed in soils naturally rich with uranium is likely the consequence of long-term exposure to high amounts of toxic metal and consecutive development of a uranium-tolerant community.

Our data also demonstrate that the elevated uranium concentration had a significant impact on bacterial community structures through the modification of species composition and relative abundance. For Vénachat and Villard sites, the PCA of DGGE profiles revealed the presence of uranium-specific fingerprints, with uraniferous samples clearly differentiated from their corresponding controls. The variation between uranium-rich soils and controls was greater than when soils were compared based on sampling date or sampling location.

In addition, the PCA of DGGE profiles revealed that uraniferous profiles were more dispersed in Vénachat than in Villard soils. Considering that the uranium content of soils from Villard was much higher than from Vénachat, this result suggests a possible dose effect relationship of uranium on the bacterial community structure. The PCA also illustrates that the Villard bacterial communities, subjected to high uranium exposure, were remarkably stable over time. As such, no major changes were observed between uraniferous samples collected in 2006, 2007 and 2009, whereas the controls exhibited more dispersion. The lack of significant variation between the uranium-rich samples reflects the high selective pressure of the metal on the bacterial communities. Taken together, our results provide clear evidence that uranium presence in the soil influences bacterial diversity and imposes a permanent constraint on bacterial communities.

In Villard samples, the sequencing of DGGE bands specific to uranium-rich soils revealed that 51% of the 144 sequenced clones had at least one close neighbor (>95% similarity) in different uranium-containing environments worldwide. The phylogenetic analysis of the sequences revealed an abundant population of *Acidobacteria* and *Proteobacteria*. The presence of an important population of *Acidobacteria* is not surprising since they are widely distributed in the environment and are frequently detected in polluted soils, including uranium-contaminated environments [39], [40]. The most abundant group of *Acidobacteria*-related sequences found in Villard (DGGE band n° 55) shows close relationships to sequences retrieved from an iron(II)-rich seep [33], and from uranium-contaminated environments (access. number AJ519372, AJ582043). Furthermore, sequences related to *Geothrix* (a genus of iron-reducing bacteria) were detected in the DGGE band; related sequences have also been retrieved during uranium reduction in iron-rich creek soil from a former uranium mining area [41], [42]. These results suggest that *Acidobacteria* may play an important role in iron and uranium redox reactions in the uranium-rich soils of Villard. The enrichment of Fe(III)-reducing bacteria in the uranium-rich soils of Villard was confirmed by the detection of *Geobacteraceae* in the DGGE bands. Members of this *Deltaproteobacteria* family have been shown to play an active role during U(VI) reduction in sediment [22], [42]. Likewise, several species of *Geobacter* are capable of U(VI) reduction using cytochromes involved in the anaerobic Fe(III) respiration [1], [43]. Our own results indicate that in addition to *Geobacter,* a group of *Acidobacteria* may play a significant role in uranium as well as iron reduction in soil from Villard.

Strikingly, in our study we concomitantly detected iron-oxidizing and iron-reducing species in the uranium-rich soils. Indeed, close relatives to known iron-oxidizers such as *Gallionella* and *Sideroxidans* were retrieved from the DGGE bands. *Gallionella ferruginea* produces extracellular material in the form of a spirally twisted stalk where iron can precipitate. The stalk of this bacterium has been shown to be able to accumulate metal, including uranium [44]. Recently *Gallionella* related species were reported to predominate the bacterial community during uranium reoxidation in sediments [45]. To our knowledge, relationship between *Sideroxydans* and uranium has not yet been described in the literature. However, the sequences detected in the DGGE band are closely related to a sequence retrieved from uranium-contaminated sediments (Acces. number AJ582038). The concomitant presence of both *Gallionella* and *Geobacter* has recently been associated to a tight coupling of biological iron reduction and oxidation in an iron-rich fresh water seep [33]. Based on the DGGE band sequences, we can hypothesize the presence of a uranium redox cycle in the soil mediated by a mixed population of reducers and oxidizers. The co-occurrence of both reducers and oxidizers may result in the presence of micro-sites in the soils, where a diverse range of aerobic and anaerobic-based metabolisms may occur.

## Materials and Methods

### Experimental sites and soil sampling

Soil samples were collected from the Villard and Vénachat sites, located near Bessines-sur-Gartempe, Limousin, France. This region is well-known for the presence of important natural uranium deposits.

Vénachat is located 15 km south of Bessines-sur-Gartempe. A uranium-rich area (approximately 2 m×0.5 m) was detected with a Geiger counter, 10 m from the roadside, at the foot of a granitic outcrop exhibiting a uraniferous vein. In October 2006, 8 independent samples (approximately 50 g each) were collected on the soil surface at 2 different positions in this area (U1 and U2). A series of 8 non-radioactive control samples (C) were collected in close vicinity (<10 m apart; approximately 50 g each). Each sample was homogenized by manual mixing before DNA extraction. Subsequently, U1, U2 and C samples were pooled and mixed before pH measurement, bacteria cultivation and a partial chemical analysis. In October 2007, 8 radioactive and 8 control samples (approximately 50 g each) were collected randomly from the same areas as 2006. One DNA extraction was performed for each radioactive soil sample (1 g per extraction), after which all DNA were pooled (designated as sample U-07). DNA was similarly extracted and pooled from control samples (designated as sample C-07). Soil samples were then pooled to give U-07 and C-07 samples. Subsequent bacterial cultivation, DNA extraction and in-depth mineral and chemical analyses were performed on these samples.

At Villard, 2 km northeast of Bessines-sur-Gartempe, three separate soil samplings were performed in October 2006, October 2007 and April 2009. A very restricted radioactive spot (approximately 30 cm in diameter) was detected in a ditch along a field. Different soil samples (approximately 100 g) were collected from this area: U-06 (2006), U-07 (2007) and U-09 (2009). Samples were collected at 10–20 cm depth and stored in sterile tubes. The presence of bright yellow particles was observed in these samples. Non-radioactive control samples C-06 (2006), C-07 (2007), and C1-09; C2-09; C3-09; C4-09; C5-09 (2009) were collected in close vicinity (50 cm to 5 m apart) within the ditch. Each sample was homogenized by manual mixing and pH measurements, and DNA extraction and bacteria cultivation were processed immediately after collection. Samples were then stored at 4°C until chemical analyses could be performed.

### Bacterial count and soil analysis

To estimate the number of culturable aerobic bacteria, soil sample suspensions were spread on 0.1× TSB agar plates (TSB, Difco Laboratories). The number of CFU per g of soil was measured after incubation at 30°C for two weeks.

For pH determination, 5 g of soil were mixed with 5 ml of sterile deionized water, shaken for 1 h and decanted. pH of the supernatant was measured. Elemental composition of soil samples was determined after homogenization and mineralisation by ICP-AES for major elements (NFX31-147/NF EN ISO 11885 method) and ICP-MS for traces (NFX31-147/ISO 17294-2 method). The uranium content was measured on 0.5 g of soil samples. The Total Organic Carbon (TOC) concentration was assayed by specific equipment (Leco system - NF ISO 10694-2 method).

In order to determine the amount of water-extractable uranium, soil elutriates were prepared according to the AFNOR [46] standard protocol: 2 g of dried soil (24 h at 105°C) were mixed with 20 ml of deionised water, shaken 16 h at 20°C, centrifuged at 3000 g for 15 min and the supernatant was collected; this procedure was repeated three times. Samples for chemical analyses were acidified with HNO_3_ and stored at 4°C in the dark until analysis. Uranium content was measured in each elutriate sample by ICP-MS.

### X-ray diffraction measurements

Soil samples VeC-07, VeU-07, ViC-07 and ViU-07 were finely ground using an agate mortar and pestle. X-ray diffraction (XRD) patterns were recorded with a Panalytical X'Pert Pro MPD® diffractometer mounted in the Debye–Scherrer configuration, using an elliptical mirror to obtain a high flux, parallel incident beam and an X'Celerator® detector to collect diffracted beams. Data were recorded with a monochromatic CoKαbeam (λ = 0.17889 nm) in continuous scan mode within a (3°–120°) 2θ range with steps of 0.0167° and a counting time of 260 s per step.

### Synchrotron-Based Analysis

XAS experiments were performed at the U LIII-edge (17.166 keV) on the BM30B beamline [47] of the European Synchrotron Radiation Facility, Grenoble, France. Experimental details are given in Text S1. Spectra were normalized and EXAFS oscillations were extracted using the Athena code [48]. The resulting EXAFS curves were weighted by k3 and qualitatively analyzed by comparison between spectra of samples and references. Quantitative analysis was performed in one case using simulations performed with Artemis code.

### Scanning Electron Microscopy

One gram of the soil sample ViU-09 was incubated 2 days at 30°C in 1 ml 0.1 X TSB and the resulting suspension was deposited on a lacey 300-mesh copper grid without coating. SEM observations were performed on a Zeiss Ultra 55 FEG-. The microscope was operated at 15 kV at a working distance of 7.1 mm. Images were acquired in secondary electron mode using an Everhart Thornley detector. Energy dispersive x-ray spectrometry (EDXS) analyses were performed using an EDS QUANTAX microanalyzer with Esprit, Hypermap software that allowed acquisition of x-ray maps and drift correction.

### DNA extraction and PCR-DGGE analysis

For Vénachat soil samples, DNA was extracted from 1 g aliquots using a PowerSoil™ DNA Isolation Kit (MO Bio, USA). The yield was approximately 500 ng of DNA per gram of soil. In 2006, 8 independent DNA extractions were performed for 3 series of soil samples: U1-06, U2-06 (radioactive) and C-06 (control). For each series, 5 DNA samples were pooled (samples “p”) while 3 remained independent (samples “a, b and c”) for a subsequent DGGE analysis. In 2007, 8 DNA extractions from each radioactive (U-07) and control (C-07) soil were performed and pooled before DGGE analysis.

For Villard samples, no DNA could be recovered from the radioactive samples with the PowerSoil™ kit. An alternative SDS-based extraction method was used as described by Zhou et al. [49], starting with two 10 g aliquots of soil in 2006, and 20 g in 2007 and 2009. A final DNA purification step was added using the DNeasy tissue kit (Qiagen), following the manufacturer's instructions. The yield was approximately 30 ng of DNA per gram of soil for radioactive samples and 400 ng of DNA per gram of soil for controls.

For the DGGE analysis, metagenomic DNA extracted from soil samples was used as a template for PCR amplification of 16S rRNA genes using the universal primers set fD1 and S17 (Table S1). The 1500bp fragments were then re-amplified using primers that target *Bacteria*, *Alphaproteobacteria, Betaproteobacteria, Gammaproteobacteria, Actinobacteria, Firmicutes* and *CFB* to generate DGGE-compatible fragments. Experimental conditions for PCR reactions and DGGE electrophoresis are detailed in Text S2.

### Similarity dendrograms and Principal component analysis (PCA) of DGGE profiles

DGGE data obtained from the Genetools software were converted into a table summarizing the band presence and intensity. The total intensity of bands from each lane was normalized to 100. Principal Component Analysis (PCA) was then performed with the R-software. Statistical ellipses representing 90% confidence on PCA plots were used to compare DGGE profiles.

### Phylogenetic analysis of DGGE bands

For Villard samples, DGGE bands characterizing uraniferous profiles were excised from the gel using sterile scalpel blades. DNA was eluted in sterile water at 4°C overnight then re-amplified with primers P1 and COM2. Direct sequencing of the PCR products revealed the presence of overlapping DGGE bands with heterogeneous sequences, a feature that has been reported frequently in DGGE studies [50]-[53]. The PCR products were then cloned using the TOPO PCR cloning kit (Invitrogen Life Technologies, Carlsbad, CA, USA) following the manufacturer's instructions, prior to their sequencing. Finally, a phylogenetic analysis was performed on all 144 clone sequences obtained.

In order to assign taxonomy, clone sequences were compared to the Silva 106 reference sequences (http://www.arb-silva.de/). Each clone sequence was globally aligned (Needleman-Wunsch algorithm) to each reference sequence and a percentage of similarity was calculated using a penalty of 1 for mismatch, open a gap and extend a gap. This approach is more rigorous and precise than using BLAST, which does local alignments and searches only for exact words of 7. First we looked for hits with ≥99% similarity, in which case we calculated a consensus for their taxonomy. In the event that no hit was found with ≥99% similarity, we successively lowered the threshold in a step-wise fashion to determine at what level we could assign a taxonomy. This process was repeated until an 80% threshold was reached. Thus, the range of similarity percentages we successively looked for were: 99, 98.5, 98, 97.5, 97, 96, 95, 94, 93, 92, 91, 90, 85 and 80. In a final step, a consensus taxonomy was calculated between the hits retained from the above process. For example, a clone sequence could be assigned to a defined genus if all hits share the same genus, or simply to "Bacteria" if the selected hits conflicted at the phylum level. The sequences for each best hit from Silva were retrieved and aligned with the clone sequences using Muscle [54]. A first tree was built using conserved domains, and sequences were reordered as they occur in this tree using SeaView [55]. Alignments were checked and manually adjusted when required. Domains common to all sequences, and which show no evident sign of homoplasy, were selected and a final tree was constructed using a Kimura two parameters correction and BioNJ as implemented in SeaView.

In order to find similar sequences from polluted environments, the same similarity searches were performed with the EMBL database (release 107). In this case, every hit with greater than 95% similarity was considered valid. Each entry was analyzed for the presence of a series of keywords (uranium, radionuclide and iron). Finally the tree was displayed using [56] and information retrieved as described above was used to build Figure S2.

### Nucleotide sequence accession numbers

The 16S rRNA gene sequences determined in this study were deposited in GenBank under Accession No. JN032139–JN032282.

## Supporting Information

Figure S1 **k^3^-weighted EXAFS curve of the precipitates extracted from Villard soils.** (A): Extracted EXAFS oscillations. Experimental data (solid line) is fit (dotted line) using the parameters described in Table 1. (B): Fourier transform (FT) of the extracted EXAFS oscillations, with its magnitude (solid line), imaginary part (dotted line) and real part (dashed line). R: bond length.
(TIF)

Figure S2 **Unrooted phylogenetic tree of partial bacterial 16S rRNA gene sequences.** This tree includes every 144 sequences from DGGE bands and the most similar sequences retrieved from the public database Silva.
(TIF)

Table S1 **Phylogenetic affiliation of 16S bacterial sequences.** The sequences derived from DGGE bands characterizing uranium-rich soil samples from Villard. The number of close relatives (having >95% sequence similarity), detected in uranium-contaminated or iron-rich environments is indicated in the two last columns.
(PDF)

Text S1 **Synchrotron-Based Analysis.**
(DOC)

Text S2 **PCR amplification and DGGE analysis.**
(DOC)
