# Supplementary figures and images for: Influence of Uranium on Bacterial Communities: A Comparison of Natural Uranium-Rich Soils with Controls
Source: PLoS One. 2011 Oct 5;6(10):e25771. doi: 10.1371/journal.pone.0025771 (PMC3187815; doi:10.1371/journal.pone.0025771)

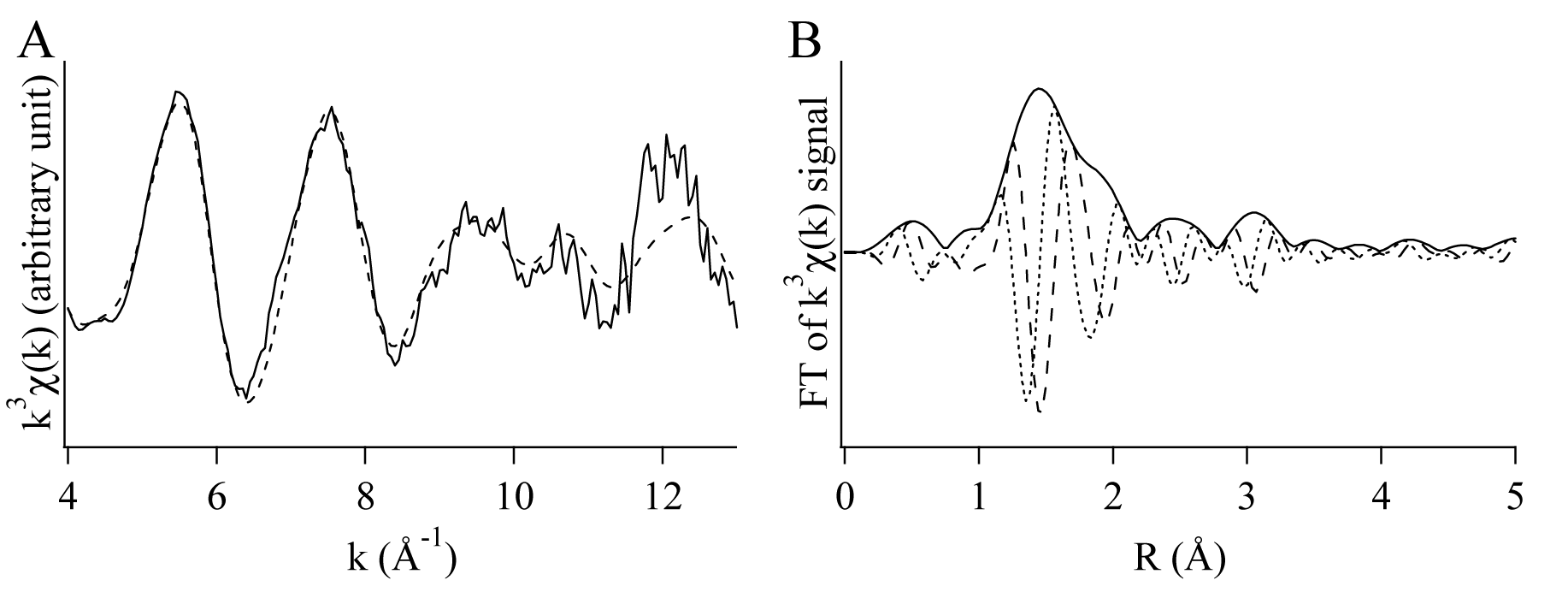

Supplement: Figure S1 — k3-weighted EXAFS curve of the precipitates extracted from Villard soils. (A): Extracted EXAFS oscillations. Experimental data (solid line) is fit (dotted line) using the parameters described in Table 1. (B): Fourier transform (FT) of the extracted EXAFS oscillations, with its magnitude (solid line), imaginary part (dotted line) and real part (dashed line). R: bond length. (TIF) [file pone.0025771.s001.tif]

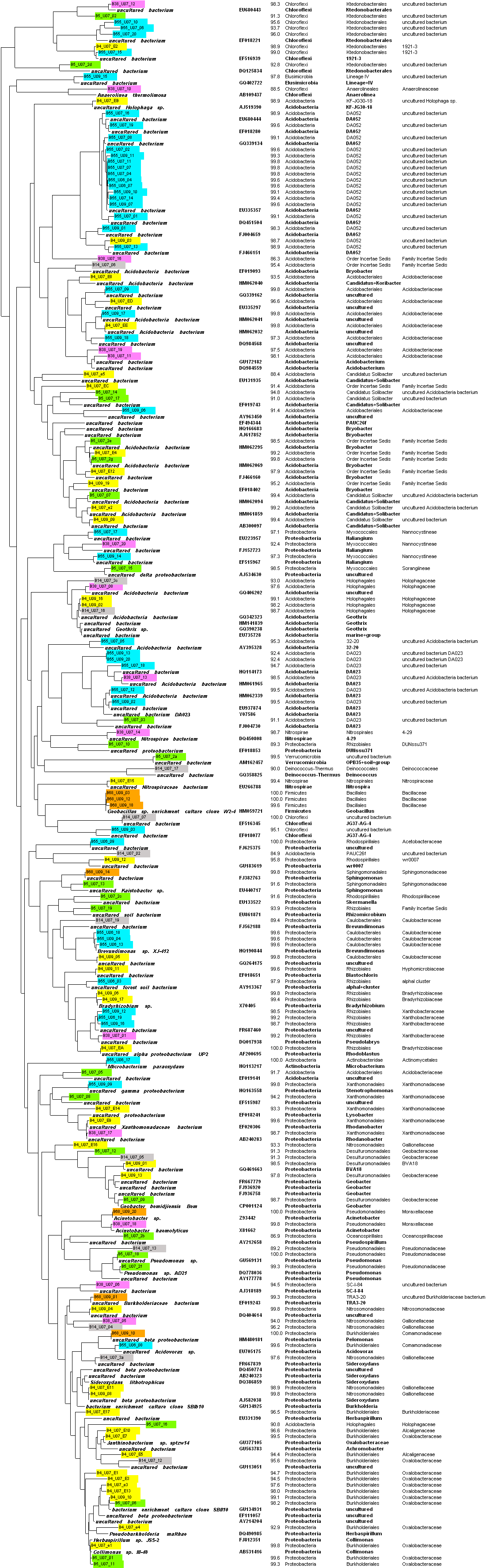

Supplement: Figure S2 — Unrooted phylogenetic tree of partial bacterial 16S rRNA gene sequences. This tree includes every 144 sequences from DGGE bands and the most similar sequences retrieved from the public database Silva. (TIF) [file pone.0025771.s002.tif]
