# Supplementary material for: Influence of Uranium on Bacterial Communities: A Comparison of Natural Uranium-Rich Soils with Controls
Source: PLoS One. 2011 Oct 5;6(10):e25771. doi: 10.1371/journal.pone.0025771 (PMC3187815; doi:10.1371/journal.pone.0025771)
Supplement: Table S1 — Phylogenetic affiliation of 16S bacterial sequences. The sequences derived from DGGE bands characterizing uranium-rich soil samples from Villard. The number of close relatives (having >95% sequence similarity), detected in uranium-contaminated or iron-rich environments is indicated in the two last columns. (PDF) [file pone.0025771.s003.pdf]

	Closest relatives (Silva 104)					Number of neighbours >95% in EMBL database	
DGGE band	similarity (%)	Accession number	Taxonomy			with uranium	with iron
B14_U07_19	89.4	FJ562188	Alphaproteobacteria	Caulobacteraceae	uncultured bacterium	-	-
B4_U09_05	99.8	GQ264175	Alphaproteobacteria	Caulobacteraceae	uncultured bacterium	8	2
B55_U06_13; 18; B55_U09_04	99.6	HQ190844	Alphaproteobacteria	Caulobacteraceae	Brevundilonas sp. XJ-412	12	5
B5_U07_10	89.3	EF018853	Alphaproteobacteria	DUNssu371	uncultured bacterium	-	-
B55_U06_03	97.9	AY913367	Alphaproteobacteria	alphaI cluster	uncultured forest soil bacterium	-	1
B4_U07_EA	100	AF200695	Alphaproteobacteria	Bradyrhizobiaceae	uncultured bacterium	2	2
B4_U09_06; 17	99.8-99.4	X70405	Alphaproteobacteria	Bradyrhizobiaceae	Bradyrhizobium sp.	19	22
B4_U09_11	99.6	EF018651	Alphaproteobacteria	Hyphomicrobiaceae	uncultured bacterium	-	-
B5_U07_19	93.9	EU861871	Alphaproteobacteria	Rhizobiaceae	uncultured soil bacterium	-	-
B38_U07_01	99.2	DQ017938	Alphaproteobacteria	Xanthobacteraceae	uncultured bacterium	20	8
B55_U09_12;16; B55_U06_19	99.2-98.5	FR687460	Alphaproteobacteria	Xanthobacteraceae	uncultured bacterium	26	11
B55_U06_09	100	FJ625375	Alphaproteobacteria	Acetobacteraceae	uncultured bacterium	-	2
B5_U07_2c	91.6	EU133522	Alphaproteobacteria	Rhodospirillaceae	uncultured bacterium	-	-
B14_U07_02; B4_U09_12	95.8-80	GU183619	Alphaproteobacteria	wr0007	uncultured bacterium	-	-
B5_U07_13	91.6	EU440717	Alphaproteobacteria	Sphingomonadaceae	uncultured Kaistobacter sp.	-	-
B68_U09_14	99.8	FJ382763	Alphaproteobacteria	Sphingomonadaceae	uncultured bacterium	22	4
B4_U07_E17	96.5	GU134925	Betaproteobacteria	Burkholderiaceae	bact. enrichment culture clone SBlB10	2	3
B68_U09_10	100	HM480181	Betaproteobacteria	Comamonadaceae	uncultured beta proteobacterium	3	19
B55_U06_08	99.6	EU705175	Betaproteobacteria	Comamonadaceae	uncultured Acidovorax sp.	10	61
B4_U07_e4	92.9	DQ490985	Betaproteobacteria	Oxalobacteraceae	Pseudoburkholderia malthae	-	-
B4_U07_E7; E10	99.5-96.6	GU377105	Betaproteobacteria	Oxalobacteraceae	Janthinobacterium sp. Sptzw13	18	2
B4_U07_E1; E3; E13; e3; B4_U09_10	99.1-94.5	GU134931	Betaproteobacteria	Oxalobacteraceae	bact. enrichment culture clone SBN10	19	5
B5_U07_06	98.2	GU134931	Betaproteobacteria	Oxalobacteraceae	bact. enrichment culture clone SBN15	3	2
B4_U07_E5; B14_U07_12	95.6-94.4	GU113051	Betaproteobacteria	Oxalobacteraceae	uncultured bacterium	-	-
B5_U07_01; 11; B4_U07_e1	99.8-99.3	AB531416	Betaproteobacteria	Oxalobacteraceae	Collimonas sp. III-48	18	9
B68_U09_01	99.3	EF019243	Betaproteobacteria	TRA3-20	uncultured bacterium	2	4
B14_U07_3a	97.5	FR667839	Betaproteobacteria	Gallionellaceae/Sideroxydans	uncultured bacterium	-	6
B4_U07_E11	98.9	AJ582038	Betaproteobacteria	Gallionellaceae/Sideroxydans	uncultured bacterium	1	74
B4_U09_08	99.8	AJ582038	Betaproteobacteria	Gallionellaceae/Sideroxydans	uncultured bacterium	2	76
B38_U07_05	94	AB240323	Betaproteobacteria	Gallionellaceae	uncultured bacterium	-	-
B14_U07_04	96.2	AB240324	Betaproteobacteria	Gallionellaceae	uncultured bacterium	-	17
B4_U07_E16	93.3	DQ450774	Betaproteobacteria	Gallionellaceae	uncultured bacterium	-	-
B4_U09_04	99.8	DQ4O4614	Betaproteobacteria	Nitrosomonadaceae	uncultured bacterium	5	12
B38_U07_06	94.5	AJ318189	Betaproteobacteria	SC-I-84	uncultured bacterium	-	-
B68_U09_20	100	Z93442	Gammaproteobacteria	Moraxellaceae	Acinetobacter sp.	14	16
B38_U07_18	99.8	X81662	Gammaproteobacteria	Moraxellaceae	Acinetobacter haemolyticus	11	14
B5_U07_2f	99.3	DQ778036	Gammaproteobacteria	Pseudomonadaceae	Pseudomonas sp.AD21	71	82
B5_U07_18	100	GU569131	Gammaproteobacteria	Pseudomonadaceae	uncultured Pseudomonas sp.	63	81
B14_U07_13	89.2	GU569132	Gammaproteobacteria	Pseudomonadaceae	uncultured Pseudomonas sp.	-	-
B5_U07_2b	86.9	AY212658	Gammaproteobacteria	Oceanospirillaceae	uncultured bacterium	-	-
B55_U09_09	99.8	HQ163558	Gammaproteobacteria	Xanthomonadaceae	uncultured bacterium	8	1
B4_U07_E14	93.3	EF018241	Gammaproteobacteria	Xanthomonadaceae	uncultured bacterium	-	-
B5_U07_08	94.2	EF515987	Gammaproteobacteria	Xanthomonadaceae	uncultured bacterium	-	-
B4_U07_E9	99.6	EF020306	Gammaproteobacteria	Xanthomonadaceae	uncultured bacterium	4	2
B38_U07_17	98.7	AB240283	Gammaproteobacteria	Xanthomonadaceae	uncultured bacterium	4	2
B5_U07_12	91.3	GU270815	Deltaproteobacteria	Geobacteraceae	uncultured bacterium	-	-
B14_U07_05	91.3	AY607189	Deltaproteobacteria	Geobacteraceae	uncultured Geobacter	-	-
B4_U09_13	97.8	FR667779	Deltaproteobacteria	Geobacteraceae	uncultured bacterium	9	17
B5_U07_09	98.7	CP001124	Deltaproteobacteria	Geobacteraceae	Geobacter bemidjiensis	10	6
B4_U09_01	98.5	GQ461663	Deltaproteobacteria	BVA18	uncultured bacterium	4	22
B5_U07_15	98.5	AJ534630	Deltaproteobacteria	Sorangiineae	uncultured bacterium	1	-
B55_U07_17	97.1	EU223957	Deltaproteobacteria	Nannocystineae	uncultured bacterium	4	2
B38_U07_20	92.4	FJ152723	Deltaproteobacteria	Nannocystineae	uncultured bacterium	-	-
B55_U09_14	97.3	EF515967	Deltaproteobacteria	Nannocystineae	uncultured bacterium	-	-

B4_U07_E12	97.5	FJ466160	Acidobacteria	Family Incertae Sedis	Bryobacter	6	1
B4_U09_19	95.2	EF018402	Acidobacteria	Family Incertae Sedis	Bryobacter	-	-
B5_U07_2e; 2g	99.8-98.5	HM062295	Acidobacteria	Family Incertae Sedis	Bryobacter	5	1
B4_U07_E4	99.2	HM062295	Acidobacteria	Family Incertae Sedis	Bryobacter	5	1
B14_U07_06	95.4	EF019093	Acidobacteria	Family Incertae Sedis	Bryobacter	-	-
B38_U07_16	86.3	FJ206864	Acidobacteria	Family Incertae Sedis	uncultured bacterium	-	-
B4_U07_EC	91.4	FM956224	Acidobacteria	Family Incertae Sedis	uncultured bacterium	-	-
B55_U07_09	99.8	GQ339162	Acidobacteria	Acidobacteriaceae	uncultured bacterium	3	1
B55_U09_06	91.4	AY963450	Acidobacteria	Acidobacteriaceae	uncultured bacterium	-	-
B55_U09_17	99.8	HM062041	Acidobacteria	Acidobacteriaceae	uncultured bacterium	4	-
B55_U09_18	97.3	DQ984568	Acidobacteria	Acidobacteriaceae	uncultured bacterium	-	-
B4_U07_E8	93.5	HM062040	Acidobacteria	Candidatus Koribacter	uncultured bacterium	-	-
B4_U07_ED	96.6	EU335297	Acidobacteria	Acidobacteriaceae	uncultured bacterium	-	-
B4_U07_EE	99.8	HM062032	Acidobacteria	Acidobacteriaceae	uncultured bacterium	4	-
B38_U07_11	98.1	GU172182	Acidobacteria	Acidobacteriaceae	uncultured bacterium	-	2
B38_U07_19	97.5	DQ984559	Acidobacteria	Acidobacteriaceae	uncultured bacterium	-	2
B5_U07_05	91.7	EF019141	Acidobacteria	Acidobacteriaceae	uncultured bacterium	-	-
B4_U07_e5	88.4	EU131935	Acidobacteria	Candidatus Solibacter	uncultured bacterium	-	-
B5_U07_14; 17	94.8-91	EF019743	Acidobacteria	Candidatus Solibacter	uncultured bacterium	-	-
B4_U09_09	99.4	AB300097	Acidobacteria	Candidatus Solibacter	uncultured bacterium	1	3
B4_U07_e2	99.2	HM061859	Acidobacteria	Candidatus Solibacter	uncultured bacterium	1	2
B5_U07_07	99.4	HM062094	Acidobacteria	Candidatus Solibacter	uncultured bacterium	-	2
B55_U07_18	94.7	HQ114173	Acidobacteria	DA023	uncultured bacterium	-	-
B55_U09_02	99.5	EU937874	Acidobacteria	DA023	uncultured bacterium	17	4
B55_U07_12	99.5	HM062339	Acidobacteria	DA023	uncultured bacterium	-	-
B55_U09_13; 20	92.3	EU297423	Acidobacteria	DA023	uncultured bacterium	-	-
B5_U07_03	91.1	FJ004730	Acidobacteria	DA023	uncultured bacterium	-	-
B38_U07_13	98.5	HM061965	Acidobacteria	DA023	uncultured bacterium	-	-
B55_U07_13; B4_U09_03	98.9-98.7	FJ466151	Acidobacteria	DA052	uncultured bacterium	2	-
B55_U07_02; 04; 07; 08; 11; 14	99.8-99.1	EU335357	Acidobacteria	DA052	uncultured bacterium	6	2
B55_U09_07; 10; 11	99.6-99.1	EU335357	Acidobacteria	DA052	uncultured bacterium	6	2
B55_U06_04; 07	99.6	EU335357	Acidobacteria	DA052	uncultured bacterium	6	2
B55_U09_01	98.3	FJ004659	Acidobacteria	DA052	uncultured bacterium	-	-
B55_U07_01	99.1	DQ451504	Acidobacteria	DA052	uncultured bacterium	5	2
B55_U07_16	98.9	EU680444	Acidobacteria	DA052	uncultured bacterium	7	2
B55_U07_19	99.6	EF018280	Acidobacteria	DA052	uncultured bacterium	6	2
B4_U07_EB	98.9	AJ519390	Acidobacteria	KF-JG30-18	uncultured Holophaga	6	-
B55_U07_05	95.3	AY395328	Acidobacteria	32-20	uncultured bacterium	-	-
B38_U07_08	97.6	GQ406202	Acidobacteria	Holophagaceae	uncultured bacterium	-	1
B14_U07_3c	93	GQ406202	Acidobacteria	Holophagaceae	uncultured bacterium	-	-
B4_U09_02; 16	99.1-98.2	GQ342323	Acidobacteria	Holophagaceae/Geothrix	uncultured bacterium	8	4
B14_U07_16	98.7	GQ342323	Acidobacteria	Holophagaceae/Geothrix	uncultured bacterium	8	6
B5_U07_16	90.8	GU205678	Acidobacteria	Holophagaceae	uncultured bacterium	-	-
B55_U07_06; 10; 20	93.7-96	EF018221	Chloroflexi	Ktedonobacterales	uncultured bacterium	-	-
B38_U07_12	98.3	EU680443	Chloroflexi	Ktedonobacterales	uncultured bacterium	17	4
B5_U07_02	91.3	EU680443	Chloroflexi	Ktedonobacterales	uncultured bacterium	-	-
B5_U07_2d	92.8	DQ125834	Chloroflexi	Ktedonobacterales	uncultured bacterium	-	-
B4_U07_E2; B55_U07_15	99-98.9	EF516939	Chloroflexi	1921-3	uncultured bacterium	-	-
B38_U07_10	88.5	AB109437	Chloroflexi	Anaerolineaceae	Anaerolinea thermolisa	-	-
B55_U09_03	95.1	EF018077	Chloroflexi	JG37-AG-4	uncultured bacterium	-	-
B14_U07_07	100	EF516345	Chloroflexi	JG37-AG-4	uncultured bacterium	3	-
B68_U09_03; 12; 18	100-99.6	HM059721	Firmicutes	Bacillaceae	Geobacillus sp.	-	-
B4_U07_E15	99.4	EU266788	Nitrospirae	Nitrospiraceae	uncultured Nitrospirae bacterium	5	12
B38_U07_14	98.7	DQ450808	Nitrospirae	4_29	uncultured Nitrospirae bacterium	1	3
B55_U06_17	100	HQ113217	Actinobacteria	Microbacteriaceae	Microbacterium paraoxydans	17	4
B14_U07_17	90	GQ358825	Deinococcus-Thermus	Deinococcaceae	uncultured bacterium	-	-
B55_U09_15	97.8	GQ402722	Elusimicrobia	Lineage IV	uncultured bacterium	-	-
B5_U07_2a	99.5	AM162457	Verrucomicrobia	OPB35 soil group	uncultured bacterium	-	-
