# Supplementary material for: Influence of Uranium on Bacterial Communities: A Comparison of Natural Uranium-Rich Soils with Controls
Source: PLoS One. 2011 Oct 5;6(10):e25771. doi: 10.1371/journal.pone.0025771 (PMC3187815; doi:10.1371/journal.pone.0025771)
Supplement: Text S1 — Synchrotron-Based Analysis. (DOC) [file pone.0025771.s004.doc]

**Text S1: Synchrotron-Based Analysis**

XAS experiments were performed at the U L_III_-edge (17.166 keV) on the BM30B beamline (Proux *et al.*, 2005) of the European Synchrotron Radiation Facility, Grenoble, France. The storage ring was operated in 16 bunches mode at 6 GeV with a ~90 mA current. The beam energy was selected using a Si(220) double-crystal monochromator with an experimental resolution close to the theoretically predicted value (~0.9 eV) (Proux *et al*, 2006). The beam size on the sample was approximately 300 x 200 µm (H x V). Spectra were recorded in fluorescence mode for the samples and liquid references, using a 30-element solid state Ge detector (Canberra, Olen, Belgium), and in transmission mode for one solid sample, pressed as a pellet (U-phosphate). The monochromator was energy-calibrated with a metallic Zr foil (17998 eV).

Spectra were normalized and EXAFS oscillations were extracted using the Athena code (Ravel and Newville 2005). The resulting EXAFS curves were weighted by k^3^ and qualitatively analyzed by comparison between the spectra of samples and references. Quantitative analysis was performed in one case using simulations performed with Artemis code.

Proux O, Biquard X, Lahera E, Menthonnex JJ, Prat A et al. (2005) FAME: a new beamline for x-ray absorption investigations of very-diluted systems of environmental, material and biological interests. Phys Scr 970.

Proux O, Nassif V, Prat A, Ulrich O, Lahera E et al. (2006) Feedback system of a liquid nitrogen cooled double-crystal monochromator : design and performances. *J. Synchrotron Radiation* 13: 59-68.

Ravel B, Newville M (2005) ATHENA, ARTEMIS, HEPHAESTUS: data analysis for X-ray absorption spectroscopy using IFEFFIT. *J. Synchrotron Radiation* 12: 537-541.
