# Supplementary material for: Influence of Uranium on Bacterial Communities: A Comparison of Natural Uranium-Rich Soils with Controls
Source: PLoS One. 2011 Oct 5;6(10):e25771. doi: 10.1371/journal.pone.0025771 (PMC3187815; doi:10.1371/journal.pone.0025771)
Supplement: Text S2 — PCR amplification and DGGE analysis. (DOC) [file pone.0025771.s005.doc]

**Text S2: PCR amplification and DGGE analysis**

For PCR-DGGE bacterial community analysis, we used a nested approach. In a first step, metagenomic DNA extracted from soil samples was used as a template for a PCR amplification of 16S rRNA genes using the universal primers set fD1 (5’-AGAGTTTGATCCTGGCTCAG-3’) and S17 (5’-GTTACCTTGTTACGACTT-3’) as described previously (Chanal *et al*., 2006). The 1500pb fragments were then re-amplified using P1-GC (5'‑CGCCCGCCGCGCGCGGCGGGCGGGGCGGGGGCACGGGGGG'CCTACGGGAGGCAGCAG‑3) and COM2 (5’-CCGTCAATTCCTTTGAGTTT-3’) to generate DGGE compatible fragments (Muyzer et al., 1993; Schwieger et al., 1998). The PCR amplification reaction mixture (50 μl) contained 1× PCR buffer, 0.2 mM of each dNTP, 0.5 μM of each primer, 20 ng of template DNA and 2.5 U of DyNAzyme EXT Taq DNA polymerase (FINNZYMES, Finland). After initial denaturation (94°C for 5 min), products were amplified for 30 cycles with the following program: 94°C for 30 s, 55°C for 30 s and 72°C for a duration based on the primer set used (1 min per kb), followed by a final extension (5 min, 72°C). For DGGE analysis, PCR products generated from each sample were applied to 10% (w/v) polyacrylamide-bisacrylamide (37.5:1) gels prepared in 1x TAE buffer with different linear denaturant gradients (100% denaturant equals 40% (v/v) formamide and 7M urea) depending on the fragments to be separated. Electrophoresis was performed in 1x TAE at 120 V and 60°C for 18 h using the DCode Universal Mutation System (Bio-Rad Laboratories, France). DGGE gels were stained either with silver nitrate or with SYBR Green, and high resolution gels images were captured in a G:BOX System using the GeneSnap software (SYNGENE, England). DGGE profiles were analysed by the software Genetools (SYNGENE, England) to obtain densitometric curves and to create a matrix for the PCA analysis.

Chanal A, Chapon V, Benzerara K, Barakat M, Christen R, et al. (2006) The desert of tataouine: an extreme environment that hosts a wide diversity of microorganisms and radiotolerant bacteria. Environ Microbiol 8: 514-525.

Muyzer G, De Waal EC, Uitterlinden AG (1993) Profiling of complex microbial populations by denaturing gradient gel electrophoresis analysis of polymerase chain reaction-amplified genes coding for 16S rRNA. Appl Environ Microbiol 59: 695-700.

Schwieger F, Tebbe CC (1998) A new approach to utilize PCR-single-strand conformation polymorphism for 16S rRNA gene-based microbial community analysis. Appl Environ Microbiol 64: 4870-4876.
